# CILIA2012: Cilia in development and disease

**DOI:** 10.1186/2046-2530-1-S1-I1

**Published:** 2012-11-16

**Authors:** Hannah M Mitchison, Philip L Beales

**Affiliations:** 1 Molecular Medicine Unit and Birth Defects Research Centre, University College London, Institute of Child Health, 30 Guildford Street, London WC1N 1EH, UK

## 

In May 2012, around 270 researchers from 20 different countries participated in the CILIA2012 conference held at the UCL Institute of Child Health, in London. This was the first international scientific conference devoted to Cilia in Development and Disease co-organised with the Ciliopathy Alliance, a consortium of ciliopathy patient organisations. These proceedings provide an almost complete account of the scientific content of the meeting and contribute to describing the current state of cilia research during an exciting period for this rapidly expanding field.

The last decade has seen spectacular and rapid advances in our understanding of the ubiquity and central role for cilia in developmental processes, and our insight into the molecular basis of numerous diseases that have emerged as 'ciliopathies'. In recognition of the broad sweep of interests amongst the conference delegates and within the field, the conference sessions covered five mains subject areas. These with the keynote speakers who presented in each session were: (1) Clinical and novel aspects of ciliopathies (Heymut Omran, University of Münster, Germany and Friedhelm Hildebrandt, University of Michigan, USA); (2) Structure and function of cilia (Brad Yoder, University of Alabama, USA and Greg Pazour, University of Massachusetts, USA); (3) Cilia and development (Kathryn Anderson, Sloan-Kettering Institute, USA and Jeremy Reiter, University of California San Francisco, USA); (4) Cilia and disease (Enza Maria Valente University of Messina, Italy, and Nicholas Katsanis, Duke University, USA); (5) Translational therapy and ciliotherapeutics (Peter Jackson, Genentech and Rachel Giles, University Medical Center Utrecht, Netherlands).

A concentrated schedule of talks and posters chosen from the submitted abstracts was assembled by the organising committee which included significant unpublished data. Thirty-nine oral presentations were given, and 139 posters presented in total, covering many different diseases, numerous model organism systems, and including studies of almost all organs in the body. The plenary speaker was John Wallingford, (University of Texas at Austin, USA) who spoke on 'Planar cell polarity, cilia and human disease'.

The scientific congress was preceded by a public engagement event that provided a unique opportunity for clinical and scientific researchers to meet with patients and relatives who suffer from ciliopathies, and other organisations working in their support. Joseph Gleeson (University of California San Diego, USA) gave the keynote talk at this event 'Translating gene discoveries for patient benefit'. Information stands were well attended by participants, organised by a number of groups including EuroWABB, Polycystic Kidney Disease Charity, Primary Ciliary Dyskinesia Family Support Group, Laurence Moon Bardet Biedl Society, Joubert Syndrome in the UK, Kidney Research UK, Deafness Research UK and the Genetic Alliance/Rare Disease UK.

## Acknowledgments

We would like to acknowledge the major support of the following sponsors and exhibitors who made the meeting possible, and for which we are very grateful: SYSCILIA, an EU FP7 systems biology consortium; Company of Biologists; the Genetics Society; BioMed Central - CILIA journal; EuroWABB; Kidney Research UK; Deafness Research UK, Roche; Perkin Elmer and Mammalian Genome. Tess Harris administered the meeting with impeccable skill and good humour.

For more information on the the Ciliopathy Alliance and the science discussed at the conference, visit http://www.ciliopathyalliance.org/.

